# *Mycobacterium Avium* complex vertebral osteomyelitis in the absence of HIV infection: a case report and review

**DOI:** 10.1186/s12879-018-3143-4

**Published:** 2018-05-22

**Authors:** Megan E. Gray, Peter W. Liu, Brian Wispelwey

**Affiliations:** 1 Division of Infectious Diseases and International Health, University of Virginia Health System, P.O. Box 801379, Charlottesville, VA 22908-1361 USA; 2 Department of Infectious Diseases, University of Pennsylvania Health System|, 3400 Spruce Street, Philadelphia, PA 19104 USA

**Keywords:** Mycobacterium avium complex, Non-tuberculous mycobacteria, Vertebral osteomyelitis, Chronic corticosteroid use

## Abstract

**Background:**

*Mycobacterium Avium* Complex (MAC) is an established microbiologic cause of pulmonary disease, lymphadenitis, and disseminated disease in cases of advanced immune suppression. However, MAC manifesting as vertebral osteomyelitis is less common, and is particularly rare in the absence of Acquired Immunodeficiency Syndrome (AIDS). Prompt diagnosis of MAC vertebral osteomyelitis is challenging, but necessary to prevent serious morbidity or mortality.

**Case presentation:**

We report a case of MAC osteomyelitis of the lumbar spine in a 70-year-old woman on extended duration corticosteroid therapy for systemic lupus erythematosus who presented with progressive back pain. Upon presentation, imaging revealed osteomyelitis of the lumbar spine with associated paraspinal abscess. Cultures from the surgical evacuation of the paraspinal abscess yielded no pathogen growth and she was therefore treated with empiric antibacterial therapy. Two weeks after her initial hospital discharge she represented with severe back pain and radiologic evidence of progressive disease in her lumbar spine. Two additional vertebral biopsies were required during her first 2 weeks of admission. MAC eventually grew from culture 14 days after collection. She was treated with ethambutol and rifampin and her symptoms resolved in 2 weeks, though therapy was continued for 12 months.

**Conclusions:**

MAC is an unusual cause of vertebral osteomyelitis in patients with AIDS, but is exceedingly rare in those without severe immune compromise. Despite its rarity, it must be considered in cases of vertebral osteomyelitis that do not respond to empiric antibiotic therapy. Multiple biopsies may be necessary to obtain a diagnosis and avoid destructive infectious complications of an untreated infection.

## Background

*Mycobacterium Avium* Complex (MAC) includes two important human pathogens, *Mycobacterium avium* and *Mycobacterium intracellulare.* MAC organisms are ubiquitous in the environment and have been identified in typical reservoirs of soil, water, and animals [1]. There are no documented cases of horizontal transmission of non-tuberculous mycobacteria (NTM) infections [2], and manifestations of human disease arise most commonly after acquisition of bacterial inoculum via inhalation or ingestion [1, 3].

The most well recognized disease manifestations of MAC are pulmonary disease, lymphadenitis, and in the case of advanced immunocompromised, disseminated disease [1, 3, 4]. Yet, of considerable importance to the clinician are also less common presentations of MAC, such as osteomyelitis. Although bacterial vertebral osteomyelitis is not difficult to diagnose, delayed recognition of MAC as the causative pathogen is likely to lead to significant adverse sequelae. Particularly vexing are difficulties with rapid microbiologic identification owing to the fastidious characteristics of MAC organisms and treatment challenges that arise from the complexity and duration of recommended therapies.

Vertebral osteomyelitis due to MAC is a poorly described entity. The majority of cases in the medical literature are presented in hosts affected by Human Immunodeficiency Virus (HIV) or AIDS [4–8], though there are reports of MAC osteomyelitis in HIV infected patients with immune reconstitution and CD4 T-lymphocyte counts well above the recommended threshold for maintenance of MAC prophylaxis [8]. Here we report a case of lumbar vertebral osteomyelitis due to MAC in a patient on systemic corticosteroid therapy and review the available literature regarding this rare infection.

## Case presentation

A 70-year-old woman presented with 5 months of progressive low back pain. She had a complex medical history including a remote splenectomy, anti-phospholipid syndrome, autoimmune hemolytic anemia requiring previous courses of cyclophosphamide, L3 laminectomy 2 years prior, and systemic lupus erythematosus (SLE) with ongoing therapy with hydroxychloroquine and prednisone 20 mg daily. Her low back pain initially manifested in the context of a herpes zoster infection and management of presumed neuropathic pain was pursued. In ensuing months, she had progression of low back pain despite conservative management. Magnetic Resonance Imaging (MRI) of the lumbar spine showed evidence of an epidural abscess at L2–3, L3–4 with vertebral osteomyelitis at L2-L3. Admission vitals showed a heart rate of 120 beats per minute, blood pressure of 121/59 mmHg, temperature of 36.9 °C, respiratory rate 16 breaths per minute, and SpO2 of 93% on room air. She had a mild leukocytosis with a white blood cell count of 12.18 k/uL. C-reactive protein and sedimentation rate were elevated at 2.8 mg/dL and 45 mm/h respectively. She underwent surgical evacuation of the epidural abscess and wound revision of L2-L3. Intra-operative findings included dark brown fluid that egressed from her epidural site, but no purulent fluid was visualized. Intraoperative cultures of vertebral bodies and discs showed no evidence of bacterial or fungal growth. Acid-fast bacterial (AFB) stains, cultures and QuantiFERON®-TB Gold In-Tube testing were also negative. Empiric treatment with vancomycin and cefepime was initiated with plans to complete a six-week course.

Three weeks after surgical intervention, she re-presented with persistent low back pain and encephalopathy. Cefepime was considered as a possible etiology of her altered mental status and therefore was replaced by aztreonam. Admission vital signs were unremarkable. C-reactive protein and sedimentation rate were 4.9 mg/dL and 36 mm/h respectively, with notable increase in C-reactive protein from 2.8 mg/dL. Lumbar spinal computed tomography (CT) demonstrated severe lytic and sclerotic destructive changes centered on the disc space of L2-L3 and the vertebral body of L4. There were also findings consistent with a large paraspinal abscess anterior to the L3 vertebral body. A biopsy of the L3 vertebral body was obtained and showed no organisms on gram stain with no growth after 7 days. This prompted a repeat bone biopsy in attempts to define the causative pathogen and direct further antimicrobial therapy. Meanwhile, empiric antibiotics to cover typical pathogens were continued. Two weeks following the initial vertebral body biopsy there was growth of acid fast bacilli from the bony specimen, which was further identified as MAC by hybridization probe. In vitro susceptibility testing indicated a favorable resistance profile with susceptibility to clofazimine, rifabutin, clarithromycin, ethambutol, and rifampin. Treatment with ethambutol and azithromycin was initiated after receiving susceptibility results. Two-drug therapy was selected due to the favorable susceptibility of the MAC isolate, in addition to efforts to mitigate potential adverse drug effects and medication interactions. She had no evidence of MAC pulmonary involvement on chest CT and the etiology of her infection was uncertain. Two months later she was seen in follow-up with resolution of her symptoms. She will continue antibiotics to complete a 12-month course.

## Discussion

There have been 16 case reports describing MAC causing vertebral osteomyelitis in non-HIV infected individuals in the literature to date (Table 1). As discussed, HIV/AIDS is a risk factor for MAC infection as are other forms of immunosuppression related to medications or genetic defects. In patients without known immune compromise, additional factors that may heighten clinical suspicion for MAC vertebral osteomyelitis include advanced age, osteoporosis, trauma, previous spinal surgery, or known pulmonary disease [9]. Of the reported cases, including the patient discussed in this report, 53% (*n* = 9) had no known immunodeficiency, 47% (*n* = 8) were on chronic corticosteroid therapy, 18% (*n* = 3) had osteoporosis and 12% (*n* = 2) were surgically asplenic. Osteoporosis is not thought to be a risk factor due to direct pathophysiologic influence, but rather the potential for physicians to attribute patients’ symptoms and associated radiologic findings to compression fractures with resultant delay in directed therapy.

**Table 1:** Summary of clinical details from published cases of MAC vertebral osteomyelitis in patients without a known HIV diagnosis

REFERENCE	Country	Age, SEX	Clinical manifestations	Underlying Conditions	Site of Involvement	Antibacterial/ Months of Therapy	Surgical Intervention	Diagnosis Method/ Weeks to Positivity	Biopsies
9	U.S.A.	72, F	Mid-thoracic back pain, fevers, LE* weakness, decreased sensation	Polymyositis, steroid use	T11-L1 osteomyelitis, extra-dural soft tissue mass compressing anterior spinal cord	clarithromycin, ethambutol, rifampin/NA	No	(Acid fast stain lead to TB diagnosis) Tissue culture/ NA	1
10	U.S.A.	62, F	Low back pain	None	Paraspinal abscess at L5-S1, destructive changes of L5 and S1 vertebral bodies.	clarithromycin, clofazamine, ethambutol/21	Yes	Tissue culture/NA	1
11	U.S.A.	35, M	Right shoulder pain	SLE, steroid use	Right humeral head osteomyelitis, thoracolumbar infection with associated paraspinal abscess	ethambutol, isoniazid, streptomycin/24+, until death	Yes	Tissue culture/NA	1
12	Japan	76, F	MAC pulmonary infection, low back pain	None	T4-T5 osteomyelitis	clarithromycin, moxifloxacin, rifampicin/NA	Yes	Tissue culture/NA	1
13	U.S.A.	79, M	Low back pain, urinary incontinence	SLE, steroid use, osteoporosis	Thoracolumbar osetomyelitis with spinal cord compression due to soft tissue paraspinal mass. 10 cm lung mass with pleural effusion.	Initial empiric: Isoniazid, rifampin, pyrazinamide/2.25 Definitive: amikacin/4, clindamycin, clofazamine, ethambutol, rifampin/6 - until death	Yes	(Acid fast stain lead to TB diagnosis) Tissue culture/9	2
17	Japan	38, F	Low back pain	SLE, steroid use	Septic arthritis of bilateral knees, T8-T9 paravertebral abscess and T9 osteomyelitis	clarithromycin, ethambutol, rifampin/12	Yes	Tissue culture/NA	1
17	Japan	50, M	Low back pain	None	Paravertebral abscess and psoas abscess at level of L4, osteodiscitis at L2-L5.	clarithromycin, ethambutol, rifampin/+ 12	Yes	Tissue culture/5	1
18	China	60, M	Low back pain	None	L2-L3 osteomyelitis with psoas abscess	Initial empiric: ethambutol, isoniazid, pyrazinamide, rifampicin/3. Definitive: amikacin/2, ethambutol, rifampicin/12	Yes	(Pathology lead to TB diagnosis) Tissue culture/12	1
19	Japan	67, M	Low back pain, fever	Diabetes mellitus	T spine to L spine with psoas abscess	clarithromycin, cycloserine, ethambutol, rifampicin, streptomycin sulfate/+ 24	Yes	PCR* from sinus tract/8	1
20	Australia	70, F	Low back pain, paraplegia	Osteoporosis, chronic bronchitis	T12 osteomyelitis with spinal cord compression	clarithromycin, ethambutol, rifampin/NA	No	Tissue culture/2	2
21	U.S.A.	60, F	Low back pain, fevers, productive cough	Sarcoidosis, steroid use, COPD, osteoporosis, splenectomy	T7-T9 osteomyelitis with paraspinal abscess causing cord compression at T8-T9.	Initial empiric: clarithromycin, ethambutol, isoniazid, rifampin. Definitive: clarithromycin, ethambutol, rifampin/24	Yes	(Acid-fast stain lead to TB diagnosis) Tissue culture/NA	1
26	U.S.A.	27, F	Low back pain, fevers, LE weakness and hyporeflexia	SLE, steroid, hydroxychloroquine use	L5 osteomyelitis, soft tissue mass extending into spinal canal and anteriorly into retroperitoneum, 1 cm breast nodule	cycloserine, ethambutol, rifampin, streptomycin/20 months – until death.	Yes	Tissue culture from breast/NA	1
27	U.S.A.	39, M	Low back pain, paraplegia	None	T6 and T7 destruction, paraspinal abscess with spinal cord impingement.	ciprofloxacin, erythromycin, ethambutol/6	Yes	Tissue culture/NA	1
28	U.S.A.	31, F	Back pain, painful adenopathy	None	Axillary abscess, sternal abscess, T7-T8 osteomyelitis with paraspinal abscess, metastatic osteomyelitis to proximal femurs, pelvis, sternum, and distal radius	Initial empiric: clarithromycin, isoniazid, pyridoxine, rifampin Definitive: ciprofloxacin, clarithromycin, cycloserine, ethambutol, rifampin/24	No	(Acid-fast stain from abscesses lead to TB diagnosis) Tissue culture/NA	1
29	U.S.A.	12, F	Left leg pain	None	Multifocal osteomyelitis involving the tibias, right femur, pelvis, spine and orbit	NA	Yes	Tissue culture/NA	1
30	Australia	70, M	Back pain, ataxia	ILD, steroid use	T5-T7 osteomyelitis with vertebral destruction	NA	Yes	Tissue culture/NA	1

*F* female, *M* male, *LE* lower extremity, *SLE* systemic lupus erythematosus, *COPD* chronic obstructive pulmonary disease, *ILD* interstitial lung disease, *NA* data not available, *TB Mycobacterium tuberculosis*, *PCR* polymerase chain reaction

Identifying the mode of transmission in patients with MAC infection is difficult, and there is often no discernable exposure history. Several case reports cite prior trauma as the risk factor for development of infection [10, 11], whereas others were thought to have a pulmonary MAC infection with subsequent hematogenous seeding of the spine [12, 13]. Another theory that has been proposed is that of *locus minoris resistentiae*, (Latin for “place of less resistance”), a longstanding medical concept that a weakened part of the body may be more susceptible to disease [10, 14]. In this case, the area of the patient’s spine that was manipulated during preceding laminectomy could have predisposed her to infection and seeding from her subsequent steroid injection. Likewise, the steroid injection may have caused local trauma leading to potential inoculation of MAC from another unknown source. Contamination of air conditioning systems, surgical tools or materials, tissue-marking agents or colonized aqueous solutions with NTM have all been observed and should be considered as potential exposures in patients who have had medical procedures or surgeries [3].

Early diagnosis of MAC infection is a challenge due to the slow growing nature of mycobacteria. Up to 6 weeks of incubation time is often required for cultures to show evidence of growth. Regardless of the causative pathogen, vertebral osteomyelitis is a diagnostic challenge. Even in patients with pyogenic osteomyelitis only 28% of cases are diagnosed within the first month of symptom onset [15]. Diagnosis of osteomyelitis can be made without image guided aspiration biopsy or open biopsy in patients with positive blood cultures for typical causative organisms, such as Staphylococcus or Brucella [16]. Generally, blood cultures are negative and a bone or soft tissue biopsy for histopathological and microbiological diagnosis is required. Bone and soft tissue cultures may also be unrevealing and repeat biopsies are recommended, but not always performed given the risks associated with invasive procedures [16]. Of the five case reports that discussed the time elapsed between biopsy and identification of the causative pathogen, the time course ranged from 2 to 12 weeks [13, 17–20]. Additionally, 18% (*n* = 3) of cases required more than one biopsy to establish a diagnosis [13, 19, 20].

Our patient had culture negative vertebral osteomyelitis when first diagnosed. She had no risk factors for mycobacterium infection, apart from chronic corticosteroid use. The first two bone and soft tissue biopsies obtained were not immediately helpful in establishing any microbiologic diagnosis. Multidisciplinary discussion and collaboration led to a third bone biopsy, which is the recommended course of action. Due to the slow growth of MAC, it was the second bone biopsy that showed growth, 2 weeks after it was collected. This highlights the need for repeated biopsies in patients with culture negative vertebral osteomyelitis, particularly when accompanied by a poor clinical response to empiric therapy.

Additional recommended methods for diagnosing MAC vertebral osteomyelitis include histologic evaluation [16]. In mycobacterial infection, microscopic evaluation of tissue biopsies may show infiltrating histiocytes and granulomatous change, but these findings are neither sensitive nor specific [19]. Several of the reported MAC vertebral osteomyelitis cases were initially treated with empiric anti-tuberculous agents based on histopathologic findings of granulomatous inflammation, without additional positivity from histopathologic stains or cultures. Though empiric treatment of tuberculosis is recommended in cases with evidence of mycobacterial infection, in these cases it has led to increased morbidity given MAC’s inherent resistance of many anti-tuberculosis agents [9, 13, 18, 21]. As AFB stains do not differentiate between tuberculous and non-tuberculous mycobacteria, the addition of empiric therapy for NTM infections in patients with positive AFB stains should be considered [21].

Therapeutic options for MAC vertebral osteomyelitis include both surgical and medical interventions. MAC vertebral osteomyelitis can often lead to abscess formation and/or fistulous tracts, likely due to delay in diagnosis and definitive treatment. Indications for surgical debridement include abscess formation, progressive destruction of vertebral bodies or neurologic compromise. There are no consensus guidelines established for the treatment of MAC skin, soft tissue or skeletal disease [2, 15]. Tailoring treatment based on in vitro susceptibility testing is recommended and is associated with a favorable clinical response [22].

It is well accepted that the macrolide is the backbone of MAC therapy, though a multidrug regimen is required as monotherapy has been shown to increase resistance [2, 23]. Clarithromycin and azithromycin have both proven to be effective in combination regimens, though clarithromycin did show more rapid clearance in patients with bacteremia [2]. Ethambutol is the generally the second recommended agent. A rifamycin is frequently added as a third agent and may have some modest benefit, though existing clinical data is limited. Rifabutin is preferred over rifampin due to superior in vitro activity against MAC. In cases of macrolide resistance, an aminoglycoside in combination with a respiratory fluoroquinolone is generally used as a replacement. Clofazimine has been associated with increased mortality in patients with disseminated MAC [24].

All pharmacotherapies have potential associated adverse effects. While macrolides are generally well-tolerated, use of ethambutol can lead to serious optic neuropathies. The rifamycins are inducers of the cytochrome P-450 oxidative enzymes and the P-glycoprotein transport system. This results in drug-drug interactions with agents such as warfarin, oral contraceptives, itraconazole, and protease inhibitors, which is often a challenge given the frequency of NTM infections in HIV infected patients [25]. Due to the limited chemotherapeutic options, the treatment regimen and duration of treatment should be tailored to each patient’s individual needs. Of the 14 case reports that discussed antimicrobial therapy, the average number of antimicrobial agents used after diagnosis of MAC was 3.4. More than three agents were often implemented in the setting of drug resistance.

Our patient was treated with a two-drug regimen for 12 months with excellent clinical response. A two-drug regimen was pursued due to the high risk for harmful drug interactions with rifamycins. In pulmonary MAC infection, treatment is continued until two negative sputum cultures are obtained [2]. However, obtaining repeat tissue samples in order to evaluate for cure in patients with vertebral osteomyelitis requires an additional invasive and potentially harmful procedure, and is therefore avoided. Of the 11 case reports that discussed length of therapy, the average duration was 16.8 months [10, 11, 13, 17–19, 21, 26–30]. Treatment course for vertebral osteomyelitis is not well established given the rarity of this disease, but at least 12 months or more is likely required.

## Conclusion

Vertebral osteomyelitis is a rare manifestation of MAC in persons with HIV or AIDS, but is even less common in persons without immunocompromising conditions. Chronic corticosteroid therapy may pose a greater risk for MAC vertebral osteomyelitis than what has previously been recognized. The diagnosis and treatment of MAC vertebral osteomyelitis is complex and challenging. Repeated attempts at tissue acquisition should be strongly considered in patients with culture negative vertebral osteomyelitis, particularly when accompanied by a poor clinical response to empiric antibiotic therapy.

## Abbreviations

AIDS: Acquired immunodeficiency syndrome
CT: Computed tomography
HIV: Human immunodeficiency virus
MAC: *Mycobacterium Avium* Complex
MRI: Magnetic resonance imaging
NTM: Non-tuberculous mycobacteria
SLE: Systemic lupus erythematosus
